# Thermal and Emission Properties of a Series of Lanthanides Complexes with *N*-Biphenyl-Alkylated-4-Pyridone Ligands: Crystal Structure of a Terbium Complex with *N*-Benzyl-4-Pyridone

**DOI:** 10.3390/molecules26072017

**Published:** 2021-04-01

**Authors:** Florentina L. Chiriac, Monica Iliş, Augustin Madalan, Doina Manaila-Maximean, Mihail Secu, Viorel Cîrcu

**Affiliations:** 1 Department of Inorganic Chemistry, University of Bucharest, 23 Dumbrava Rosie st., Sector 2, 020484 Bucharest, Romania; laura.badea88@yahoo.com (F.L.C.); monica.ilis@chimie.unibuc.ro (M.I.); augustin.madalan@chimie.unibuc.ro (A.M.); 2 National Research and Development Institute for Industrial Ecology-ECOIND, 71-73 Drumul Podul Dambovitei Str., 060652 Bucharest, Romania; 3 Department of Physics, University Politehnica of Bucharest, 313 Spl. Independentei, 060042 Bucharest, Romania; doina.manaila@physics.pub.ro; 4 National Institute of Materials Physics, P.O. Box MG-7, 077125 Magurele, Romania; msecu@infim.ro

**Keywords:** lanthanides, liquid crystals, 4-pyridone, lanthanidomesogen, emission

## Abstract

This work focuses on the investigation of the liquid crystalline behavior and luminescence properties of the lanthanide complexes of Eu(III), Sm(III) and Tb(III) with *N*-biphenyl-alkylated-4-pyridone ligands. The organic ligands having a biphenyl group attached via a long flexible spacer with either 9 or 10 carbon atoms were synthesized by the reaction between 4-hydroxypyridine and the corresponding bromide compounds. The chemical structures of the organic and lanthanide complexes were assigned based on elemental analysis, single-crystal X-ray diffraction, ^1^H, ^13^C NMR and IR spectroscopies, and thermogravimetric analysis (TGA). The X-ray diffraction analysis of a parent compound shows that the lanthanide ions are surrounded by three monodentate pyridone ligands and three bidentate nitrate ions, giving a 9-coordinate environment. The mesogenic behavior and the type of liquid crystalline phases exhibited by the new complexes were analyzed by differential scanning calorimetry (DSC) and polarizing optical microscopy (POM), and powder X-ray diffraction (XRD) studies. Only the lanthanide complexes with longer spacer (10) display a monotropic SmA phase, typically on a short thermal range (less than 10 °C). The complexes with shorter flexible chains (9) show no liquid crystalline properties with melting temperatures lower than their analogs with longer spacers. The emission spectra recorded in solid state at room temperatures show typical emission bands for each lanthanide ion employed (Eu(III), Tb(III) and Sm(III)).

## 1. Introduction

Liquid crystals based on lanthanide complexes (lanthanidomesogens) are well-investigated anisotropic materials that develop interesting photophysical properties [1,2,3,4,5,6,7,8]. Along the time, the unique photophysical properties of the lanthanide ions provided a strong fundament for the design and study of their complexes with liquid crystal properties and these compounds are mainly built around several categories of organic ligands, among the most used were the Schiff bases, β-diketonates, alkanoates, different macrocyclic ligands (phthalocyanine, porphyrin, etc.), or bis-(benzimidazolyl)pyridines [6,7]. The design of such complexes must take into account the use of suitable organic ligands that act on two different complementary perspectives: first, providing thermodynamic stability to the products and second, the capacity to exploit the antenna effect, where the organic chromophore acts as a light-harvesting antenna able to transfer the absorbed energy to the emitting levels of the lanthanide ions.

Based on these considerations, the lanthanide complexes with monodentate 4-pyridone ligands represent a recently developed class of lanthanidomesogens with several strong and appealing features, such as high thermal stability and their easily adjustable mesomorphic behavior [9,10,11,12]. These new materials’ mesogenic properties can be tuned by specifically choosing the mesogenic groups attached to the 4-pyridone ring. For example, the incorporation of the 3,4,5-tris(alkoxy)benzyl unit give columnar or lamellar phases depending on the length of the terminal alkoxy groups, while the cynobiphenyl group attached via long flexible aliphatic spacers gave lamellar phases [10]. In fact, liquid crystals based on the 4-pyridone platform have been well investigated for a long time [13,14,15,16,17,18]. Moreover, the polar 4-pyridone platform delivers several versatile functionalities: intermolecular interactions through hydrogen bonding [19,20], strong coordination properties to various metal ions [21,22,23], and last, but not least, useful intermediates to liquid crystalline methylene-1,4-dihydropyridines [14] or to ionic liquid crystals based on pyridinium salts [24,25,26,27].

The present study is dedicated to the systematic investigation of a new series of lanthanide complexes based on 4-pyridone ligands that contain one biphenyl unit attached via a flexible spacer with different lengths (9 and 10 carbon atoms). The biphenyl unit is a widely used building block for the design and preparation of calamitic liquid crystals [28,29,30,31], in particular for main chain or side-chain liquid crystalline polymers [32] and their composites (polymer dispersed liquid crystals—PDLC) [33], and, in few cases, in the construction of metallomesogens (metal complexes with liquid crystals properties) [34]. This work reports on the synthesis and characterization of new *N*-alkylated-4-pyridone ligands and their lanthanide complexes with europium(III), samarium(III), and terbium(III) ions. The X-ray diffraction analysis of the terbium(III) compound with the related *N*-benzyl-4-pyridone ligand revealed a nine-coordinate environment of the lanthanide ions. Moreover, these complexes have mesomorphic properties and exhibit a monotropic lamellar (SmA) mesophase, depending on the length of the aliphatic spacer, over a short temperature range, as supported by differential scanning calorimetry (DSC) analysis and polarizing optical microscopy (POM) observations.

## 2. Results and Discussion

**Scheme 1:**
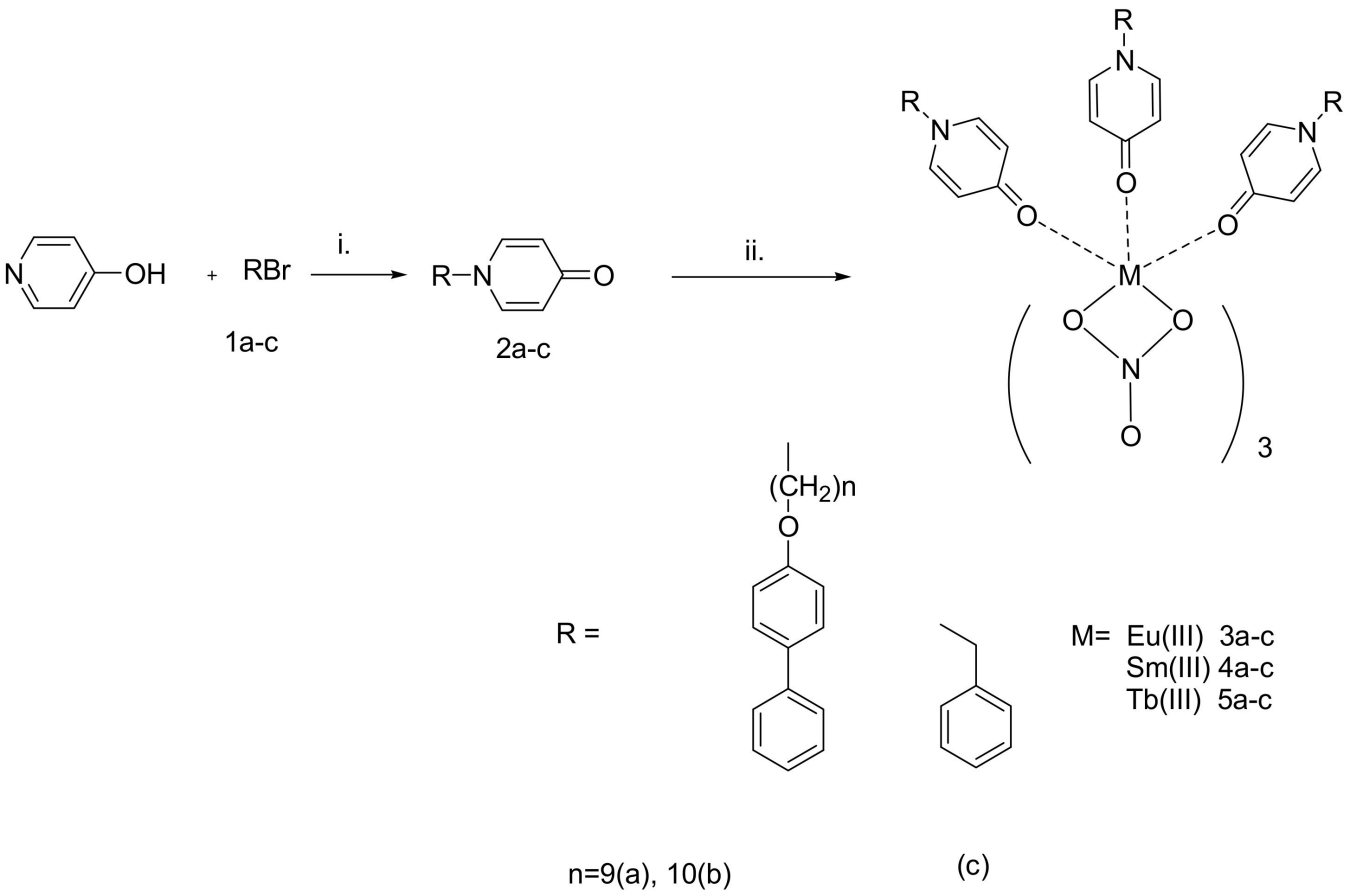
Synthetic protocol for the preparation of 4-pyridone ligands and the corresponding lanthanide complexes: (i) THF/H_2_O, NaOH 2 N, TBABr, reflux 24 h, 75 °C; (ii) M(NO_3_)_3_^.^× H_2_O (× = 5.6), EtOH, stirring 2 h at room temperature.

Recently, we have described a new class of lanthanidomesogens based on lanthanide nitrate complexes with 4-pyridone monodentate ligands, which are coordinated to the lanthanide ions via the carbonyl oxygen atom. The present study is a continuation of our investigation of this topic, lanthanidomesogens derived from 4-pyridone compounds. In this work, we report the preparation and characterization of a series of 4-pyridone ligands carrying one biphenyl group linked to the pyridone ring via a flexible spacer with 9 and 10 methylene groups and the corresponding lanthanide complexes (europium(III), samarium(III), terbium(III)). The chemical structures of the organic ligands and their complexes together with the corresponding notation are presented in Scheme 1.

### 2.1. Synthesis and Characterization of the Organic Ligands and the Lanthanide Complexes

The synthetic procedure for the 4-pyridone ligands **2a**–**c** involved the reaction between the 4-hydroxypyridine and the corresponding bromide derivative, 4-(ω-bromoalkyloxy)-biphenyl derivatives for **2a**,**b** and benzyl bromide for **2c**, in the presence of NaOH and tetrabutylammonium bromide (TBABr) as phase transfer catalyst (Scheme 1) [9,24,35].

Numerous previous reports indicated that the 4-hydroxypyridine precursors could be either *N*- or *O*-alkylated with various halide compounds giving either *N*-alkylated 4-pyridones or *O*-substituted pyridines, respectively [24,36,37,38,39].

The 4-(ω-bromoalkyloxy)-biphenyl precursors **1a**,**b** were prepared by the *O*-alkylation of 4-hydroxybiphenyl with a large excess of 1,9-dibromononane or 1,10-dibromodecane in butanone and K_2_CO_3_. The monosubstituted reaction product was isolated from the reaction mixture by repeated recrystallization from a combination of dichloromethane and ethyl ether. Finally, the organic ligands’ chemical structure and the bromide intermediates were attributed based on the results provided by the elemental analyses, ^1^H-, ^13^C-NMR and IR spectroscopies. The position of the signals assigned to the aromatic protons of the biphenyl unit is almost unchanged in the ^1^H-NMR spectra of the 4-pyridone derivatives **2a** and **2b** compare to the positions found in the ^1^H-NMR spectra of the starting bromide derivatives **1a** and **1b**. As a result of the formation of the new 4-pyridone products, the triplet signal of the BrCH_2_ group protons with the chemical shift δ = 3.42 ppm for **1a** and **1b** is replaced with the triplet signal at δ = 3.75 ppm for **2a** and **2b** assigned to the NCH_2_ protons. Several useful features could be observed in the ^13^C-NMR spectra of the 4-pyridone compounds. The aromatic region of the ^13^C-NMR spectra shows characteristic signals assigned to the carbons of the pyridone ring: δ = 178.8 ppm for the carbon atoms directly bound to the oxygen atom; δ = 139.6 ppm for the carbon atoms in the α position relative to the nitrogen atom and δ = 118.6 ppm to the carbon atoms in the β position. Moreover, the formation of the *N*-alkylated product was confirmed by the presence of a new signal at 58.9 ppm assigned to the carbon atom of the NCH_2_ group.

Several regions of interest were followed in the IR spectra of the 4-pyridone ligands in order to confirm their coordination to the lanthanide ions: the stretching vibrations assigned to the pyridone ring were observed in the 1670–1600 cm^−1^ region as very intense absorption bands; the ν(C=O) absorption bands were found at 1566 cm^−1^ for **2a** and at 1568 cm^−1^ for **2b [40]**. In the IR spectra of the lanthanide complexes, the ν(C=O) frequency is significantly shifted towards lower wavenumbers, with almost 35 cm^−1^, due to the weakening of the C=O bond resulting from the coordination of the pyridone ring to the lanthanide ion through the oxygen atom. In addition, the IR spectra of all lanthanide complexes show new characteristic absorption bands in the 1310–1320 cm^−1^ interval, assigned to the bidentate coordination of the nitrate ions providing additional strong support in favor of the proposed structure [41,42].

### 2.2. Crystal Structure of ***5c***

**Figure 1:**
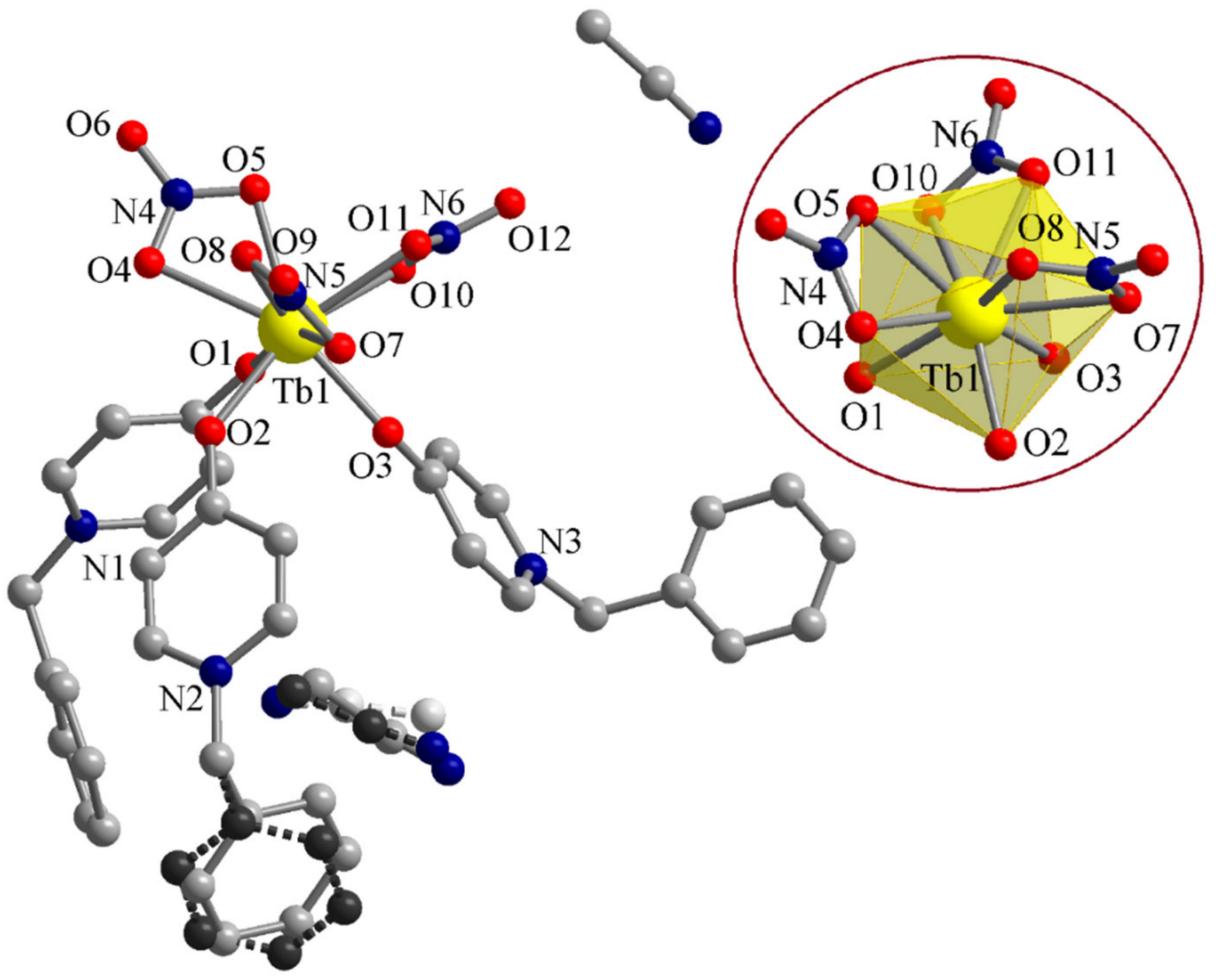
Molecular structure of the compound **5c·2CH_3_CN** (the hydrogen atoms were omitted for clarity). The phenyl ring depicted with dark gray and dotted lines represents the crystallographic position with a site occupancy factor of 0.3. The inset shows a detail of the coordination environment of the metal ion.

To get further insights into these lanthanide complexes’ molecular structure, a terbium(III) complex with a simple *N*-benzyl-4-pyridone ligand was successfully isolated as single crystals that were subjected to X-ray diffraction analysis. A summary of the crystallographic data and the structure refinement for the compound **5c**·**2CH_3_CN** are given in Table 1. This product was synthesized by following the same protocol as for the other lanthanide complexes, and the colorless block crystals suitable for X-ray diffraction were obtained from acetonitrile solution by slow evaporation. The molecular structure of the terbium(III) complex **5c** is shown in Figure 1, and the most significant bond lengths are given in Table 2. Single-crystal X-ray diffraction investigation reveals that the complex crystallizes in triclinic space group *P-1*. The compound crystallizes with two solvent molecules. One acetonitrile molecule is disordered on three crystallographic positions with site occupancy factors of 0.3333 each. These crystallization solvent molecules are lost relatively fast by exposure to air. As it is evident from the structure shown in Figure 1, the terbium(III) complex is a nine-coordinated species with the lanthanide ion surrounded by nine oxygen atoms, six oxygen atoms of the three nitrate ions coordinated in a bidentate fashion and three oxygen atoms of the three monodentate 4-pyridone ligands. The complex presents a polar distribution of the ligands around the metal ion. The inorganic ligands (the nitrate anions) are placed on one side of the metal ion, while the organic ligands are coordinating on the other side of the lanthanide. The disordered acetonitrile molecules are located in the “pocket” formed between the organic ligands due to their angular shape (Figure 1). The results of the structural analysis of the terbium(III) complex show a similar coordination sphere as found previously for the related europium(III) complex. Moreover, the Tb–O (4-pyridone) bond distances (2.262(3)–2.284(3) Å) are in the normal range and similar to the Eu–O (4-pyridone) bond distances (2.298(2)–2.318(3) Å) and to many others lanthanide complexes with carbonyl-type ligands [43,44] or pyridone-type ligands [45,46]. The slightly shorter Tb–O bond distances compare to Eu–O bond distances for the related compound, as expected, could be attributed to the lanthanide contraction. It was found that the shortest Tb–O bond distances are those toward the three 4-pyridone ligands, followed by the longer Tb–ONO_2_ bonds (2.450(3)–2.573(3) Å). In addition, the benzyl fragment of one 4-pyridone ligand (the ligand containing the oxygen atom labeled with O2 and the nitrogen labeled N2) is disordered over two crystallographic positions with site occupancy factors of 0.7 and 0.3, respectively.

**Figure 2:**
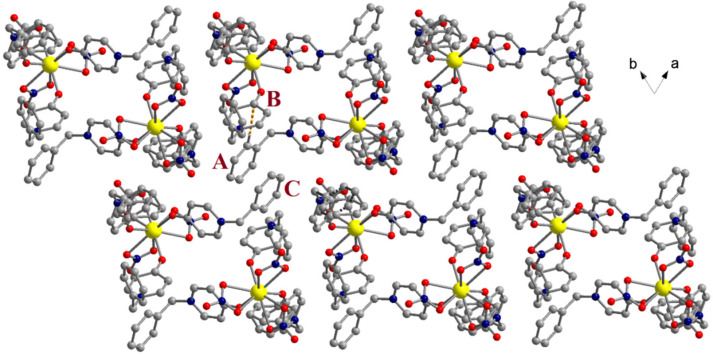
View of the packing diagram of the compound **5c** along the crystallographic *c* axis.

At the examination of the packing diagrams, CH-π and π-π interactions between neighboring molecules were evidenced in the crystallographic *ab* plane (Figure 2). The CH-π contact for the rings **A** and **B** is 2.95 Å, while the separation for the π-π interactions between the rings **A** and **C** is approximately 3.57 Å.

### 2.3. Thermal Behavior and Liquid Crystals Properties

The thermal behavior of the newly synthesized 4-pyridone organic ligands and their corresponding lanthanide complexes was investigated by thermogravimetric analysis (TGA) and differential scanning calorimetry (DSC). The nature of the liquid crystal phase was assigned based on polarizing optical microscopy (POM) observations.

**Figure 3:**
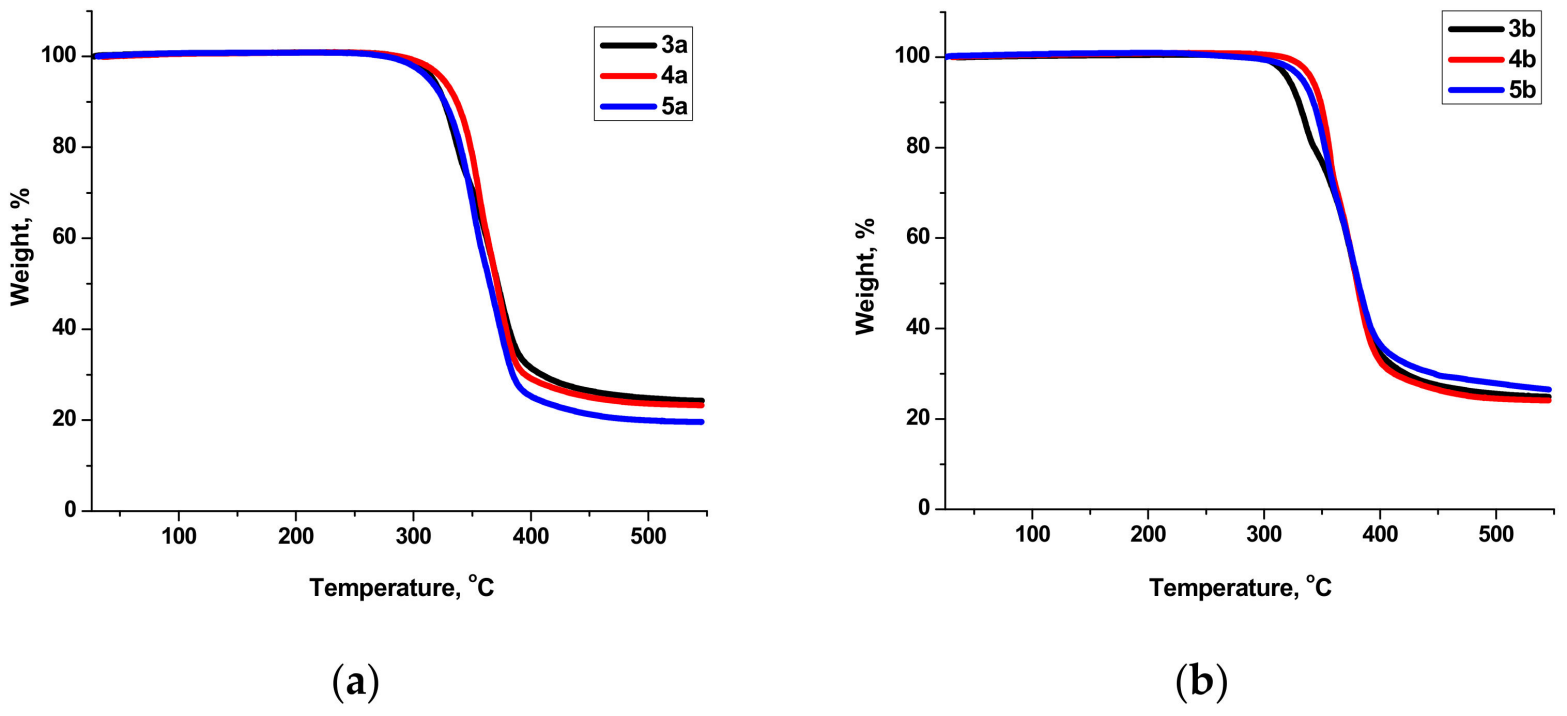

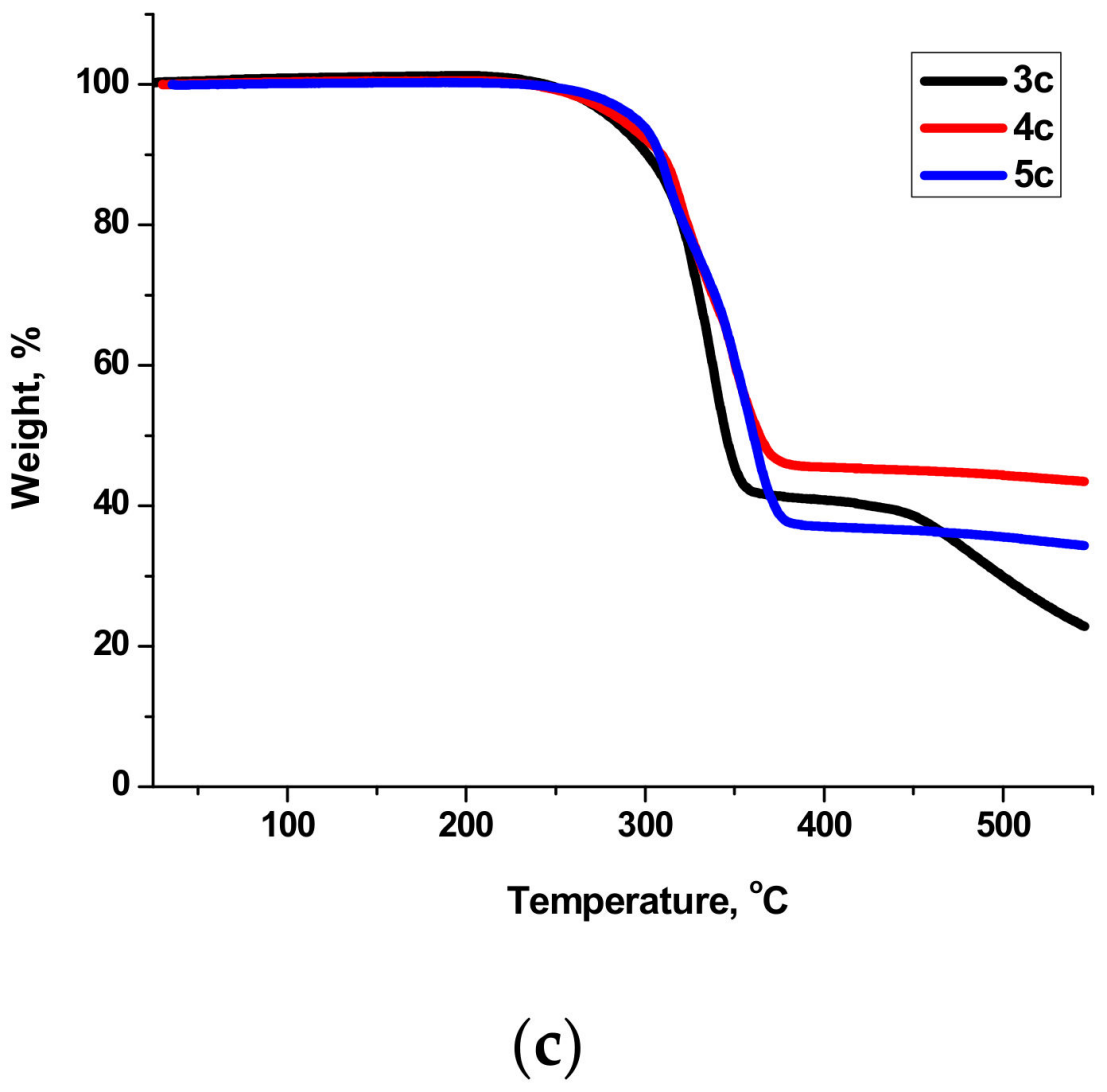
The thermogravimetric (TG) curves for the europium(III) (**a**), samarium(III) (**b**) and terbium(III) complexes (**c**).

Thermogravimetric analysis of the nine lanthanide complexes was carried out in the 25 °C–550 °C temperature range under nitrogen with a rate of 10 °C/min. The TG curves of all lanthanide complexes presented in Figure 3 show that these complexes are stable up to 300 °C, an exceptional thermal stability similar to the related lanthanide complexes with 4-pyridone ligands. Importantly, the mesogenic complexes, **3**–**5b**, exhibit a very good thermal stability extended over the whole thermal range of the liquid crystalline phase and their highest melting temperatures. In fact, during the repeating heating-cooling DSC cycles, no shifting of the transition temperatures was observed, confirming the high stability over the thermal interval used to investigate the mesomorphic properties. Moreover, the lack of any weight loss in the 25–250 °C temperature range suggests that these products have no water or any other crystallization solvent, providing strong support for the chemical structure assigned to the lanthanide complexes.

The thermal parameters, transition temperatures and the related enthalpies, measured from the second heating-cooling DSC cycles of the organic ligands and the lanthanide complexes, are collected in Table 3.

**Figure 4:**
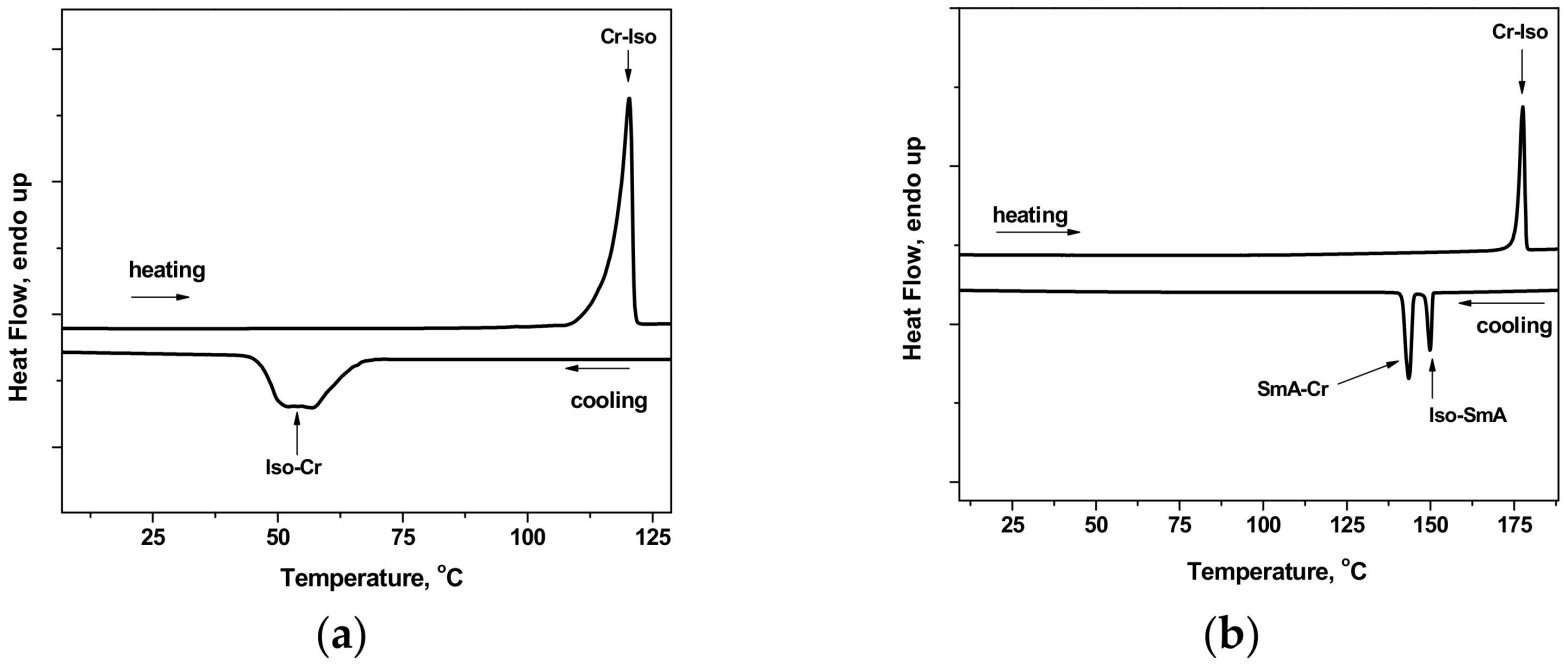
The second differential scanning calorimetry (DSC) heating–cooling cycles for compounds **2b** (**a**) and **5b** (**b**).

The three 4-pyridone ligands show no mesogenic properties, no matter the number of carbon atoms contained in the flexible spacer, and the DSC curves display only one transition during the heating or the cooling runs attributed to the melting or crystallization, respectively. In Figure 4a, the second heating-cooling DSC cycle of compound **2b** is presented as a representative example. The lack of the mesogenic properties in the case of the organic ligands was confirmed by POM observations as well when cooling the isotropic liquids resulted in the formation of the crystalline phases.

The melting temperatures of the complexes are higher on increasing the number of carbon atoms of the flexible spacer, and this trend was seen for all three lanthanides employed. The investigation of the lanthanide complexes’ thermal behavior revealed that only the complexes with longer flexible spacers (10 carbon atoms, **3**–**5b**) display a monotropic liquid crystalline phase on cooling from the isotropic phase. These phase transitions, for complexes **3**–**5b**, are easily seen both on the cooling runs of the DSC thermograms and during the POM observations. For example, the DSC thermogram of samarium(III) complex **5b** shows two distinct transitions on the cooling run (Figure 4b) and these were assigned to the isotropic to liquid crystal and liquid crystal-to-crystal phase transitions.

**Figure 5:**
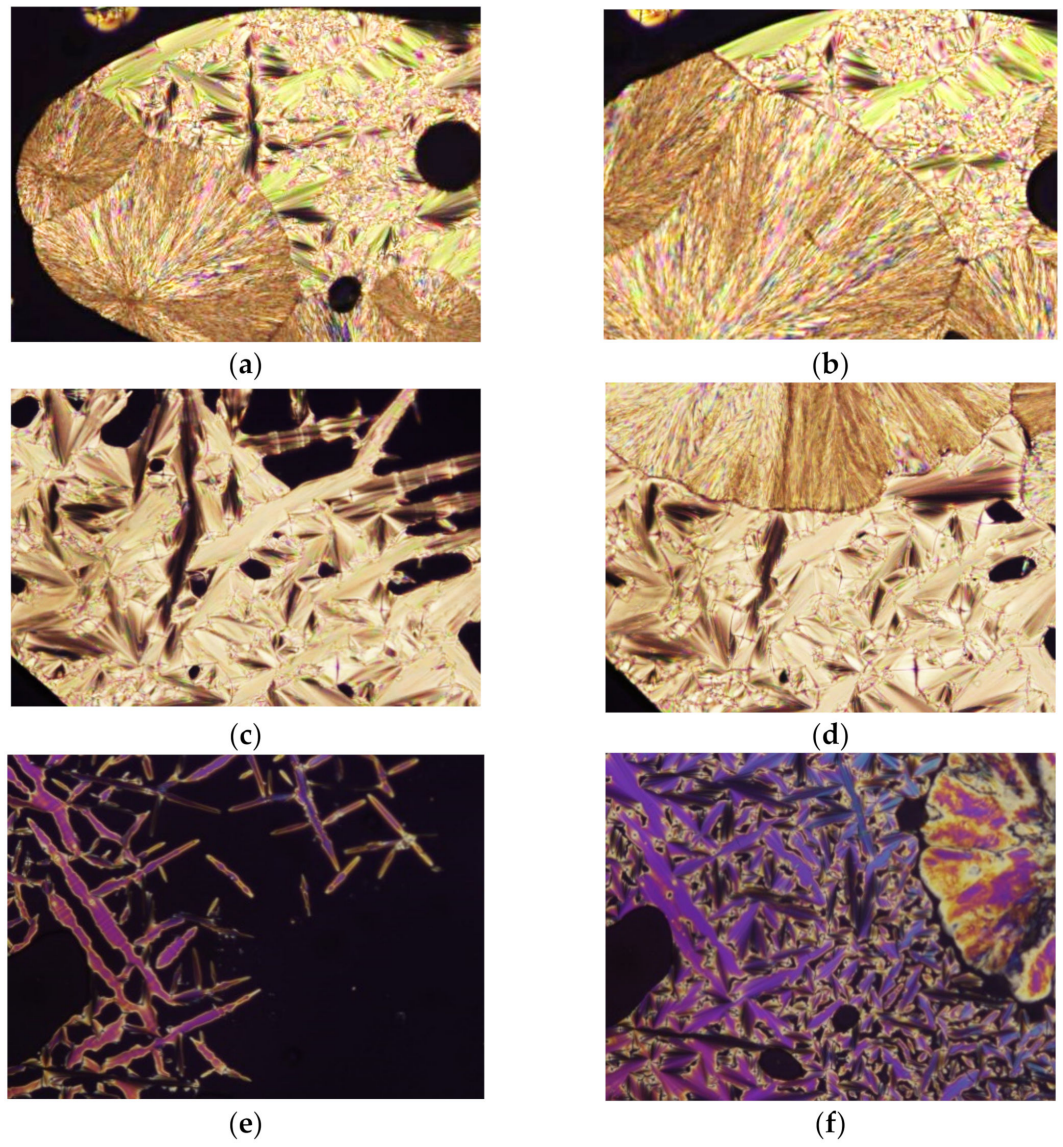
Polarized optical microscope (POM) pictures for mesogenic lanthanide complexes: **3b** at 156 °C (**a**) and at 150 °C (**b**); **4b** at 150 °C (**c**) and at 145 °C (**d**); **5b** at 155 °C (**e**) and at 150 °C (**f**).

For the other two complexes, the europium(III) **3b** and the terbium **5b** complex, the two phase transitions are too close and partially overlapped, so that it was not possible to extract the enthalpy corresponding to the isotropic to the liquid crystal phase transition. The liquid crystal phase was assigned to the SmA phase based on the POM observations. POM pictures for selected lanthanide complexes are presented in Figure 5. On cooling, the development from the isotropic phase of the texture evidenced the formation of typical “battonets” that further led to a characteristic fan-shape texture with focal-conical defects. The existence of dark homeotropic regions confirms the assignment of the SmA phase. The monotropic nature of the liquid crystal phase prevented its characterization by powder X-ray diffraction (XRD), but nevertheless, the assignment of the SmA is strongly supported by the existence of the SmA phase in the case of the analogs lanthanide complexes with 4-pyridone derivatives having the cyanobiphenyl unit instead of the biphenyl unit. In fact, the absence of the polar cyano group, by the replacement of the cyanobiphenyl unit with the biphenyl unit in the construction of the 4-pyridone ligands, is the strongest argument for the monotropic nature of the SmA phase and the lack of mesogenic properties for the analogs with shorter spacer (9 carbon atoms, complexes **3**–**5a**). Unlike the lanthanide complexes presented in this study, the related complexes with cyanobiphenyl mesogenic groups show enantiotropic SmA phases, even for shorter spacers (8, 9 or 10 carbon atoms) [11,12].

Clearly, this work demonstrates that the minimum spacer length required for stabilizing the liquid crystal phases for lanthanide complexes based on 4-pyridone ligands functionalized with biphenyl units is 10 carbon atoms, as complexes **3**–**5a** with just 9 carbon atoms in the aliphatic spacer are non-mesomorphic.

Generally, the prepared complexes’ thermal properties are similar for different lanthanide ions; the effect of changing the lanthanide ions on the thermal behavior is minimal, as previously reported for other lanthanide complexes with various 4-pyridone ligands [9,11,12].

### 2.4. Emission Properties

**Figure 6:**
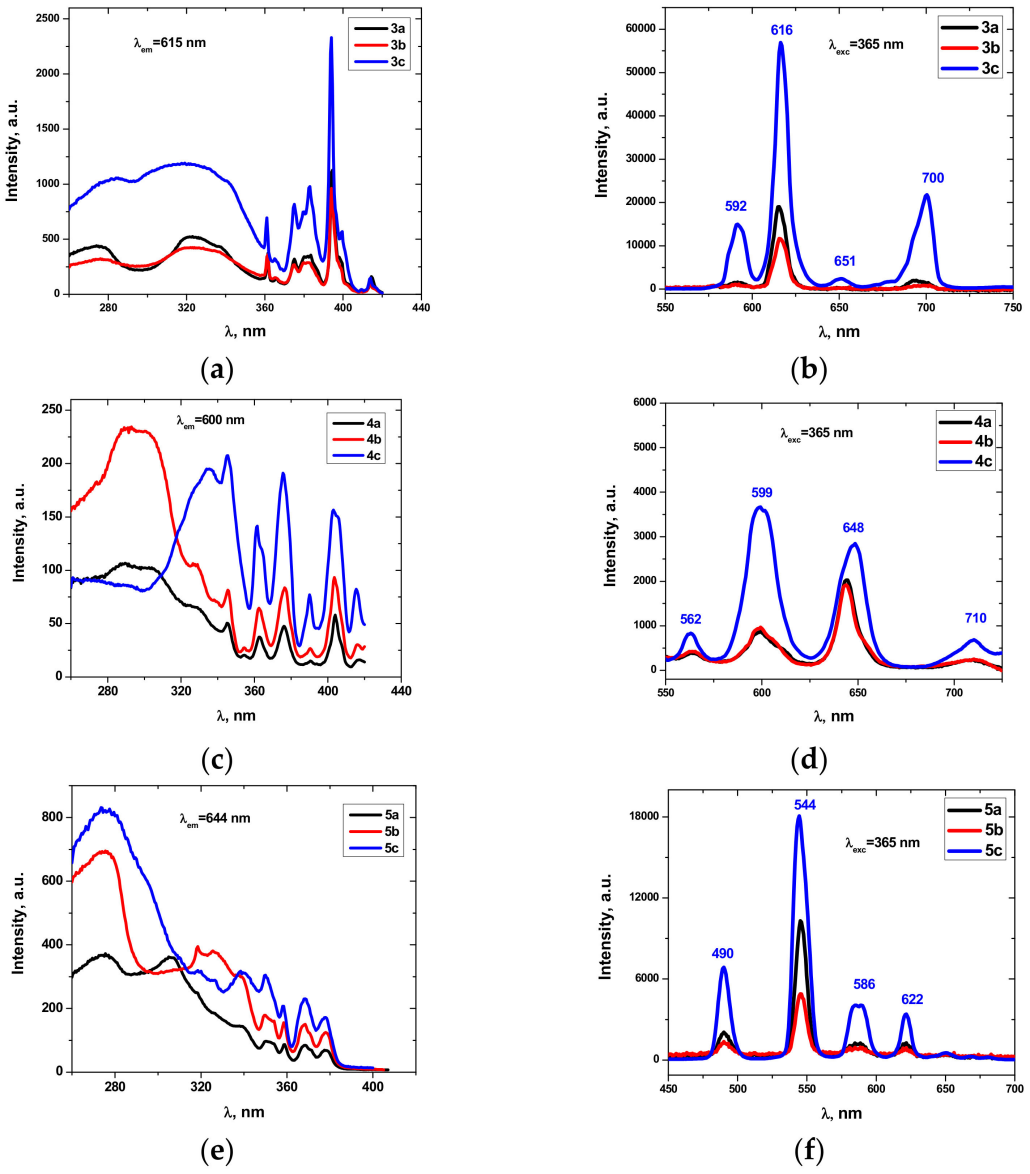
The excitation and emission spectra of the lanthanide complexes measured in solid-state at room temperature (the wavelengths used for excitation and emission are indicated on each plot): europium(III) complexes, excitation (**a**) and emission spectra (**b**); samarium(III) complexes, excitation (**c**) and emission spectra (**d**); terbium(III) complexes, excitation (**e**) and emission spectra (**f**).

Stable luminescent complexes of trivalent lanthanide ions have been long time investigated in view of their promising applications based on their exceptional emission or magnetic properties [47]. The most investigated Eu(III) and Tb(III) complexes show quite sharp and intense luminescence in the visible spectral range, red and green, respectively, while the less studied Sm(III) complexes exhibit a weaker orange emission. A correct design of the emissive lanthanide complexes must take into account the antenna effect for the sensitization of the luminescence in the lanthanide ions and, therefore, a good choice of the organic chromophores capable of absorbing light in the UV region and efficiently transferring excitation energy to the emitting levels of the lanthanide ions. The 4-pyridone based ligands are good candidates to achieve this goal, and, so far, the reported lanthanide complexes with this type of ligands showed intense luminescence properties characteristic of each type of ion employed. Liquid crystals with emissive properties have been long under scrutiny in the light of their very important applications, in particular, the polarized emission after suitable alignment [48,49,50,51]. The emission and excitation spectra for all lanthanide complexes were measured in the solid-state at room temperature and are presented in Figure 6. Two regions are easily identifiable in the luminescent excitation spectra of all lanthanide complexes: 260–350 nm, where the broad bands were assigned to the energy absorption of ligands, which can transfer energy via “ligand-to-metal” pattern, and the 350–450 nm region with very sharp peaks assigned to the absorptions of the lanthanide ions. The excitation spectra recorded for the lanthanide complexes with ligands **2a** and **2b** are similar but somehow different compared to the complexes with ligand **2c** due to the different nature of the ligands.

On the other hand, the characteristic emission bands of each lanthanide ion were observed in their emission spectra. For example, the emission spectra of the Eu(III) complexes show several bands in the 550–750 nm spectral range assigned to the ^5^D_0_ → ^7^F_J_ (*J* = 1 ÷ 4) transitions (Figure 6b). The luminescence spectra of complexes **4a**–**c**, recorded in the solid-state, show characteristic emission bands of Sm(III) ion at 562, 599, 648 and 710 nm, assigned to ^4^G_5/2_  →  ^6^H_5/2_, ^4^G_5/2_  →  ^6^H_7/2_, ^4^G_5/2_  →  ^6^H_9/2_ and ^4^G_5/2_  →  ^6^H_11/2_ transitions, respectively. Orange luminescence was observed in the solid-state for Sm(III) complexes due to the dominant ^4^G_5/2_  →  ^6^H_7/2_ transition (Figure 6d).

The spectra of the Tb(III) complexes show the transitions from the ^5^D_4_ excitation level to the ^7^F_J_ multiplets: ^5^D_4_ → ^7^F_6_ (490 nm); ^5^D_4_ → ^7^F_5_ (544 nm); ^5^D_4_ → ^7^F_4_ (586 nm); ^5^D_4_ → ^7^ F_3_ (622 nm) (Figure 6f). The samples irradiated with UV light (275 nm) emit the characteristic green light, as for all Tb(III) complexes, the ^5^D_4_ → ^7^F_5_ (544 nm) electric dipole transition is the most intense.

The decay curves for the nine samples were measured and fitted using mono-exponential function, and the luminescence lifetime values measured for europium(III) complexes with excitation at 394 nm, for samarium(III) complexes with excitation at 404 nm and for terbium(III) complexes with excitation at 375 nm are collected in Table 4. The luminescence lifetime values are characteristic for rare-earths, and the higher values observed for the complexes with ligand **2c** are consistent with the different nature of the ligands.

It is important to mention that no emission from the ligands in the emission spectra of the prepared complexes was observed. The emission spectra of the related lanthanide complexes with the 4-pyridone ligands having cyanobiphenyl terminal units in place of biphenyl units are characterized by the blue emission originating from the cyanobiphenyl group, which is dominant in the case of the Tb(III) and Sm(III) complexes [11,12]. Unlike these complexes with 4-pyridone terminated with cyanobiphenyl units, their related lanthanide complexes having the 3,4,5-tris(alkoxy)benzyl mesogenic groups attached to the 4-pyridone platform exhibited the characteristic emission light, red for Eu(III), orange for Sm(III) and green for Tb(III), respectively. For each lanthanide ion, the complexes’ emission intensities with ligand **2c** are the strongest among the three complexes of each series, and these three complexes **3**–**5c** were selected for the measurements of their quantum yields. The absolute quantum yields measured with an integrating sphere by excitation with 330 nm UV light are 7.0 ± 0.5% for **3c**, 3.0 ± 0.5% for **4c** and 16.0 ± 0.5% for **5c**, respectively.

## 3. Materials and Methods

The chemicals used in this work were purchased from Sigma-Aldrich (Saint Louis, MO, USA) and Merck (Darmstadt, Germany). The organic synthesis was monitored by TLC that was performed on commercial coated aluminum plates with Silica Gel matrix with fluorescent indicator 254 nm (Sigma-Aldrich). The visualization was done with a UV lamp (operating at 254 nm and 365 nm). C, H, N analyses were performed with a EuroEA 3300 instrumnt (Eurovector, Pavia, Italy).

A Bruker Tensor V-37 spectrophotometer (Bruker Optics Inc., Billerica, MA, USA) was employed for recording the IR spectra of all compounds in the range of 4000–400 cm^−1^. The samples were measured by ATR technique or as KBr discs. ^1^H and ^13^C NMR spectra were recorded on a Bruker Fourier 300 spectrometer (Bruker BioSpin NMR, Rheinstetten, Germany) operating at 300 MHz, using CDCl_3_ as solvent. The solvent peak positions (7.26 ppm ^1^H, 77.00 ppm ^13^C) were used to reference the ^1^H and ^13^C chemical shifts. The proposed chemical structures of the 4-pyridone compounds are consistent with the experimental data.

The liquid crystalline properties were analyzed by using a Nikon 50iPol (Nikon Instruments, Melville, NY, USA) polarized optical microscope (POM) connected with a Linkam THMS600 hot stage and TMS94 temperature control processor (Linkam Scientific Instruments Ltd., Tadworth, UK). The POM observations were done on untreated glass slides by successive heating and cooling runs. The thermal effects and enthalpies of the phase transitions were measured with a Diamond DSC PerkinElmer instrument (Perkin Elmer, Boston, MA, USA). At least two heating/cooling cycles at a scanning rate of 10 °C/min were performed for each sample, which was initially encapsulated in aluminum pans. The thermal stability of the new lanthanide complexes was checked by thermogravimetric analysis using a TA Q50 TGA instrument. The samples were placed in alumina crucibles and heated with 10 °C min^−1^ in the 25 °C to 550 °C temperature range. The measurements were done with nitrogen as purging gas. The emission properties of the lanthanide complexes were analyzed at room temperature in a solid-state. The emission spectra of the products placed on glass slides were measured with an OceanOptics QE65PRO spectrometer (Ocean Optics Inc., Orlando, FL, USA) linked via an optic fiber to the polarizing optical microscope. Two different excitation sources were used: Nikon Intensilight excitation source (Nikon Instruments) or a LED light source (LLS-LED, OceanOptics Inc., λ = 365 and 270 nm). The excitation spectra and photoluminescence decay curves were recorded at room temperature by using a FluoroMax 4P spectrophotometer (HORIBA Jobin Yvon, Kyoto, Japan). The quantum yield (QY) analysis was performed by using the Quanta-Phy accessory under 330 nm UV light excitation. X-ray diffraction measurements were performed at 200 K on an STOE IPDS II diffractometer (STOE &Cie GmbH, Darmstadt, Germany), operating with Mo-Kα (λ = 0.71073 Å) X-ray tube with graphite monochromator. The structure was solved by direct methods using SHELXS-2014 crystallographic software and refined by full-matrix least-squares techniques based on *F*^2^. The non-H atoms were refined with anisotropic displacement parameters. Calculations were performed using the SHELXL-2018 crystallographic software package. CCDC reference number: 2060438.

### 3.1. Synthesis of Intermediate Bromide Derivatives ***1a**,**b***

**Compound 1a**: 4-bromononyloxy-biphenyl compounds (**1a**) was obtained from the reaction between 4-hydroxybiphenyl (3 g, 17.6 mmol) and dibromo nonane (8.95 mL, 31.3 mmol) in the presence of K_2_CO_3_ (3 g, 21.74 mmol) and molecular sieves (3 g), in 150 mL butanone. The reaction mixture was stirred under reflux for three days in a nitrogen atmosphere. After this period, the insoluble compounds were filtered and washed with hot butanone, and the filtrate was concentrated on a rotary evaporator. The product obtained was recrystallized from DCM and ethyl ether, and the precipitate was filtered and washed with pentane. A white compound was obtained, with a yield of 81%. Yield: 81%. ^1^H-RMN (CDCl_3_, 300 MHz): 7.54 (m, 4H), 7.42 (t, 2H, *J* = 7.5 Hz), 7.30 (t, 1H, *J* = 7.3 Hz), 6.98 (d, 2H, *J* = 8.6 Hz), 4.00 (t, 2H, *J* = 6.5 Hz), 3.42 (t, 2H, *J* = 6.8 Hz), 1.96–1.71 (m, 4H), 1.47–1.25 (m, 10H). ^13^C-RMN (CDCl_3_, 75 MHz): 158.7, 133.5, 128.7, 128.1, 126.6, 114.7, 68.0, 34.1, 32.8, 29.2, 28.7, 28.1, 26.0.

**Compound 1b**: For bromide derivative synthesis (4-bromodecyloxy-biphenyl, **1b**), 4-hydroxybiphenyl (3 g, 17.6 mmol) was dissolved in 150 mL butanone, in the presence of K_2_CO_3_ (3 g, 21.74 mmol) and molecular sieves (3 g). Di-bromodecane (9.8 mL, 32.6 mmol) was added and the reaction mixture was stirred under reflux, in an inert atmosphere, for three days. The insoluble compounds were filtered, and the residue was washed with hot butanone. The solvent was removed on a rotary evaporator and the crude product was recrystallized from DCM/ethyl ether to yield a white solid. Yield 89%. ^1^H-RMN (CDCl_3_, 300 MHz): 7.54 (t, 4H, *J* = 9.6 Hz), 7.42 (t, 2H, *J* = 7.6 Hz), 7.30 (t, 1H, *J* = 7.3 Hz), 6.97 (d, 2H, *J* = 8.5 Hz), 4.14–3.88 (m, 2H), 3.42 (t, 2H, *J* = 6.8 Hz), 1.88–1.80 (m, 4H), 1.39 (dd, 12H, *J* = 30.5, 5.0 Hz). ^13^C-RMN (CDCl_3_, 75 MHz): 158.7, 140.9, 133.6, 128.7, 128.1, 126.7, 114.8, 68.0, 34.1, 32.8, 29.3, 28.7, 28.2, 26.1.

### 3.2. Synthesis of 4-Pyridone Ligands ***2a**–**c***

**Compound 2a:** A mixture of NaOH (0.389 g, 9.7 mmol in 4.86 mL of water, 2 N) and water was added to a mixture of 4-hydroxypyridine (0.924 g, 9.7 mmol) and TBABr (0.313 g, 0.94 mmol) in tetrahydrofuran (50 mL), until a clear solution was obtained. To this mixture, the halogenated derivative 1a (2.919 g, 7.8 mmol) was added and heated under reflux for 24 h. The reaction solvent was evaporated using a rotary evaporator and the solid product obtained was extracted using dichloromethane and water 1/0.8 (*v*/*v*). The organic layers were combined, dried over Na_2_SO_4_, and then the solvent evaporated to dryness. Further, the product **2a** was purified by silica gel column chromatography, using a combination of dichloromethane and methanol as eluent (95/5, *v/v*). The compound 2a was obtained as a white solid with a yield of 73%. ^1^H-RMN(CDCl_3_, 300 MHz): 7.50 (m, 6H), 7.38 (t, 2H, *J* = 7.5 Hz), 7.32–7.27 (m, 1H), 6.94 (d, 2H, *J* = 8.4 Hz), 6.35 (d, 2H, *J* = 7.4 Hz), 3.96 (t, 2H, *J* = 6.4 Hz), 3.75 (t, 2H, *J* = 6.8 Hz,), 1.85–1.55 (m, 4H), 1.50–1.19 (m, 10H). ^13^C-RMN (CDCl_3_, 75 MHz): 178.8, 158.6, 139.6, 128.6, 127.9, 126.6, 118.6, 114.6, 67.8, 58.9, 30.8, 29.4, 25.9, 24.1, 19.7, 13.6. IR (KBr, cm^−1^): 2958, 2929, 2849, 1608, 1567, 1487, 1379, 1196, 1119, 1012, 882, 839, 771, 758. **Anal. Calc. for** C_26_H_31_NO_2_: C% 80.17; H% 8.02; N% 3.60; **Found**: C% 79.44; H% 7.99; N%.54. Yield: 61%.

**Compound 2b:** Compound **2b** was obtained in a similar procedure as for **2a**, by the reaction of 4-hydroxypyridine (0.725 g, 7.6 mmol) and bromide derivative **1b** (2.38 g, 6.1 mmol) in 50 mL of tetrahydrofuran and water, in the presence of NaOH (0.305 g, 7.6 mmol in 3.82 mL water, 2 N) and TBABr (0.245 g, 0.76 mmol). The reaction mixture was heated under reflux for 24 h. After this period, the solvent was evaporated and the solid product obtained was extracted using dichloromethane and water. The organic layers were collected, dried over Na_2_SO_4_, and then the solvent was evaporated. Compound **2b** was purified by column chromatography using dichloromethane and methanol as eluent. Yield 61%, white solid. ^1^H-RMN(CDCl_3_, 300 MHz): 7.49 (m, 6H), 7.38 (t, 2H, *J* = 7.5 Hz), 7.33–7.27 (m, 1H), 6.95 (d, 2H, *J* = 8.4 Hz), 6.36 (d, 2H, *J* = 7.4 Hz), 3.97 (t, 2H, *J* = 6.4 Hz), 3.75 (t, 2H, *J* = 6.8 Hz), 1.85–1.55 (m, 4H), 1.50–1.19 (m, 12H). ^13^C-RMN (CDCl_3_, 75 MHz): 178.8, 158.6, 140.8, 133.5, 128.7, 128.1, 126.6, 117.8, 114.7, 67.9, 57.9, 30.8, 29.3, 28.9, 26.1, 25.9. IR (KBr, cm^−1^): 2923, 2850, 1609, 1566, 1485, 1391, 1175, 1115, 1012, 853, 835, 769, 721. **Anal. Calc. for** C_28_H_33_NO_2_: C% 80.36; H% 8.24; N% 3.47; **Found**: C% 79.15; H% 7.93; N% 2.24.

**Compound 2c**: Compound **2c** was obtained by adding to a solution of 4-hydroxypyridine (1.73 g, 18.2 mmol) and TBABr (0.588 g, 1.8 mmol) (phase transfer catalyst) in THF (50 mL) a 2 N aqueous solution of NaOH (0.730 g, 18.2 mmol in 1.735 mL of water). The mixture was stirred, and water was added, the solution became clear. Benzyl bromide was added to the mixture and heated under reflux for 24 h. The solvent was evaporated, and the product was extracted with dichloromethane and water. The organic layer was combined, and the solvent was removed in a vacuum; and finally, the residue was recrystallized from dichloromethane and ethyl ether. Yield 65%, white crystalline solid. ^1^H-RMN (CDCl_3_, 300 MHz): 7.38–7.32 (m, 5H), 7.19–7.16 (m, 2H), 6.40 (d, 2H, *J* = 8.5 Hz), 4.94 (s, 2H). ^13^C-RMN (CDCl_3_, 75 MHz): 178.9; 140.0; 134.7; 129.4; 129.0; 127.3; 118.9; 60.2. IR (KBr, cm^−1^): 1638, 1562, 1501, 1454, 1403, 1378, 1206, 1184, 972, 853, 731, 692, 596, 514, 483.

### 3.3. Synthesis and Analytical Data of Eu(III) Complexes ***3a**,**b***

Complexes **3a**,**b** were obtained by the dropwise addition of a solution of Eu (NO_3_)_3_·6H_2_O (0.04 mmol) in water (2 mL) over a solution of **2a**,**b** (0.12 mmol) dissolved in hot ethanol (2 mL). The reaction mixture was stirred at 65–70 °C for 2 h. **3a** and **3b** were obtained as precipitates, which were filtered and redissolved in dichloromethane, filtered through Celite and isolated by addition of ethyl ether. The synthesis of compound **3c** was reported elsewhere.

**Compound 3a:** Yield 42%. Anal. Calc. for EuC_78_H_93_N_6_O_15_: C% 62.18, H% 6.22, N% 5.58. Found: C% 62.24, H% 6.29, N% 5.49. IR (ATR, cm^−1^): 2930, 2852, 1609, 1535, 1484, 1385, 1317, 1187, 856, 833, 764, 717.

**Compound 3b:** Yield 46%. Anal. Calc. for EuC_81_H_99_N_6_O_15_: C% 62.82, H% 6.44, N% 5.43. Found: C% 62.88, H% 6.52, N% 5.41**.** IR (ATR, cm^−1^): 2925, 2852, 1610, 1531, 1380, 1320, 1186, 858, 834, 768, 718.

### 3.4. Synthesis and Analytical Data of Sm(III) Complexes ***4a**–**c***

For the synthesis of complexes **4a**–**c**, a solution of Sm(NO_3_)_3_·6H_2_O (0.04 mmol) in water (2 mL) was added dropwise over a solution of 4-pyridone ligands **2a**–**c** (0.12 mmol) in hot ethanol (2 mL) and stirred for 2 h at 65–70 °C. The compounds precipitated out of the mixture. Complexes **4a** and **4b** were isolated as white solids and purified through recrystallization from a mixture of dichloromethane and ethyl ether. Complex **4c** was filtered, washed with cold ethanol and recrystallized from hot acetonitrile.

**Compound 4a:** Yield 41%. Anal. Calc. for SmC_78_H_93_N_6_O_15_: C% 62.25, H% 6.23, N% 5.58. Found: C% 62.31, H% 6.12, N% 5.54. IR (ATR, cm^−1^): 2929, 2852, 1609, 1534, 1486, 1392, 1315, 1186, 856, 833, 717.

**Compound 4b:** Yield 47%. Anal. Calc. for SmC_81_H_99_N_6_O_15_: C% 62.89, H% 6.45, N% 5.43. Found: C% 63.05, H% 6.36, N% 5.43. IR (ATR, cm^−1^): 2925, 2852, 1610, 1531, 1468, 1391, 1319, 858, 834, 768.

**Compound 4c:** Yield 44%. Anal. Calc. for C_36_H_33_SmN_6_O_12_: %C 48.47; %H 3.73; %N 9.42; Found: %C 48.23; %H 3.88; %N 9.21. IR (KBr, cm^−1^): 1634, 1541, 1450, 1401, 1372, 1319, 1168, 1020, 819, 741, 701, 602, 500.

### 3.5. Synthesis and Analytical Data of Tb(III) Complexes ***5a**–**c***

The preparation procedure for terbium(III) complexes is similar to that used for the preparation of europium(III0 and samarium(III) complexes. Tb(NO_3_)_3_·5H_2_O (0.04 mmol) in water (2 mL) was added to a solution of 4-pyridone ligands **2a**–**c** (0.12 mmol) in ethanol (2 mL). The reaction mixture was stirred at 65–70 °C for two hours. The white precipitates formed were filtered off, redissolved in DCM and filtered over celite. The solvent was removed, and the complexes **5a** and **5b** were recrystallized from dichloromethane/ethyl ether, while the complex **5c** was recrystallized from hot acetonitrile.

**Compound 5a:** Yield: 43%. Anal. Calc. for TbC_78_H_93_N_6_O_15_: C% 61.90, H% 6.19, N% 5.55. Found: C% 62.04, H% 6.08, N% 5.37. IR (ATR, cm^−1^): 2927, 2852, 1608, 1536, 1487, 1384, 1318, 1186, 857, 836, 763.

**Compound 5b:** Yield 49%. Anal. Calc. for TbC_81_H_99_N_6_O_15_: C% 62.54, H% 6.41, N% 5.40. Found: C% 62.36, H% 6.29, N% 5.44. IR (ATR, cm^−1^): 2925, 2851, 1611, 1536, 1472, 1391, 1320, 1188, 857, 832, 765.

**Compound 5c:** Yield 47%. Anal. Calc. for C_36_H_33_TbN_6_O_12_: %C 48.01; %H 3.69; %N 9.33; Found: %C 47.88; %H 3.77; %N 9.43. IR (KBr, cm^−1^): 1634, 1543, 1450, 1401, 1373, 1317, 1168, 1021, 818, 742, 701, 602, 501.

## 4. Conclusions

This work presents the study of the thermal behavior and emission properties of the lanthanide complexes of europium(III), samarium(III) and terbium(III) with *N*-alkylated-4-pyridone ligands containing a biphenyl unit linked to the pyridone ring via a long flexible spacer with either 9 or 10 carbon atoms. For comparison purposes, the related lanthanide complexes with *N*-benzyl-4-pyridone ligand were prepared and characterized, and the terbium(III) complex was analyzed by single-crystal X-rays diffraction. The lanthanide ion is nine-coordinate and surrounded by three bidentate nitrate ions and three monodentate 4-pyridone ligands coordinated via their oxygen atoms. All complexes exhibit a very good thermal stability, and the thermogravimetric analysis shows that their decomposition starts at temperatures above 300 °C, well above the highest melting temperature of 176 °C reported for the mesogenic compounds **3b** and **4b**. On the other hand, the lanthanide complexes melting temperatures depend on the number of carbon atoms of the flexible spacer, higher temperatures for longer chains; an observation confirmed for all three lanthanides employed. The investigation of the thermal behavior of the lanthanide complexes revealed that in the absence of the polar cyano group, by the replacement of the cyanobiphenyl unit with the biphenyl unit in the construction of the 4-pyridone ligands, only the complexes with longer flexible spacer (10 carbon atoms, **3**–**5b**) display a monotropic SmA phase, while the rest of complexes are non-mesomorphic. Moreover, similar to the related lanthanide complexes with 4-pyridone ligands, the effect of changing the lanthanide ions on the thermal behavior is minimal.

These complexes show no emission originating from the organic ligands and exhibit the characteristic emission light, red for Eu(III), orange for Sm(III) and green for Tb(III), respectively, similar to their related lanthanide complexes having the 3,4,5-tris(alkoxy)benzyl mesogenic groups attached to the 4-pyridone platform. For each lanthanide ion series, the emission intensities found for complexes **3**–**5c**, with *N*-benzyl-4-pyridone ligand **2c**, are the strongest due to increased flexibility introduced by the aliphatic spacers in the molecular structure of complexes **3**–**5a**,**b**.

## Figures and Tables

**Table 1:** Crystallographic data, details of data collection and structure refinement parameters for compound **5c**·**2CH_3_CN**.

Compound	5c·2CH_3_CN
Chemical formula	C_40_H_39_N_8_O_12_Tb
*M* (g mol^−1^)	982.71
Temperature, (K)	200(2)
Wavelength, (Å)	0.71073
Crystal system	*Triclinic*
Space group	*P-1*
*a* (Å)	13.1960(8)
*b* (Å)	13.2839(8)
*c* (Å)	14.5787(10)
*α* (°)	78.722(5)
*β* (°)	80.321(5)
*γ* (°)	61.273(4)
*V* (Å^3^)	2189.6(3)
Z	2
*D*_c_ (g cm^-3^)	1.491
*µ* (mm^−1^)	1.684
F(000)	992
Goodness-of-fit on *F*^2^	1.042
Final *R1*, *wR*_2_ [*I* > 2σ(*I*)]	0.0411, 0.1039
*R1*, *wR*_2_ (all data)	0.0461, 0.1067
Largest diff. peak and hole (eÅ^−3^)	2.217, −2.174

**Table 2:** Selected bond lengths for compound **5c·2CH_3_CN**.

Tb–O (4-pyridone)	Tb1–O1 = 2.284(3); Tb1–O2 = 2.262(3); Tb1–O3 = 2.272(3)
Tb–O (NO_3_)	Tb1–O4 = 2.557(3); Tb1–O5 = 2.455(3); Tb1–O7 = 2.573(3); Tb1–O8 = 2.465(3); Tb1–O10 = 2.560(3); Tb1–O11 = 2.450(3)

**Table 3:** Thermal parameters ^(a)^ (transition temperatures in °C and the enthalpies ^(b)^ in kJ·mol^−1^) of the organic ligands and their lanthanide (III) complexes.

Compound	M	n	Transition	T (°C)	ΔH (kJ·mol^−1^)
**2a**	-	9	Cr–Iso	111	3.0
**2b**	-	10	Cr–Iso	117	45.4
**2c**	-	-	Cr–Iso	115	17.5
**3a**	Eu	9	Cr_1_–Cr_2_	135	34.3
			Cr_2_–Iso	148	46.5
**3b**	Eu	10	Cr–Iso	176	108.2
			Iso–SmA	158 ^(c)^	-
			SmA–Cr	150	104.2
**3c**	Eu	-	Cr–Iso	210	88.6
**4a**	Sm	9	Cr_1_–Cr_2_	134	1.6
			Cr_2_–Iso	145	70.6
**4b**	Sm	10	Cr–Iso	176	111.0
			Iso–SmA	151	29.5
			SmA–Cr	145	67.6
**4c**	Sm	-	Cr–Iso	217	76.7
**5a**	Tb	9	Cr_1_–Cr_2_	133	37.9
			Cr_2_–Iso	140	42.2
**5b**	Tb	10	Cr–Iso	173	92.5
			Iso–SmA	155 ^(c)^	-
			SmA–Cr	149	95.4
**5c**	Tb	-	Cr–Iso	195	83.4

^(a)^ Cr, SmA, Iso, denote crystalline, smectic A and isotropic phases, respectively. ^(b)^ These data are measured from the second heating-cooling DSC cycle; ^(c)^ The two transitions overlap, and the combined enthalpy is given.

**Table 4:** The luminescence lifetime values for the lanthanide complexes **3a**–**5c**.

Compound	M	τ (ms)
**3a**		0.716 ± 0.001
**3b**	Eu	0.719 ± 0.001
**3c**		1.493 ± 0.003
**4a**		0.055 ± 0.001
**4b**	Sm	0.055 ± 0.001
**4c**		0.071 ± 0.003
**5a**		0.807 ± 0.003
**5b**	Tb	0.624 ± 0.002
**5c**		1.260 ± 0.001

## Sample Availability

Samples of the compounds reported in this work are available from the authors.
